# Influence of Thermomechanical Treatment and Ratio of β-Lactoglobulin and α-Lactalbumin on the Denaturation and Aggregation of Highly Concentrated Whey Protein Systems

**DOI:** 10.3390/foods9091196

**Published:** 2020-08-29

**Authors:** Maria Quevedo, Ulrich Kulozik, Heike P. Karbstein, M. Azad Emin

**Affiliations:** 1 Institute of Process Engineering in Life Sciences, Chair of Food Process Engineering, Karlsruhe Institute of Technology, 76131 Karlsruhe, Germany; maria.quevedo@kit.edu (M.Q.); heike.karbstein@kit.edu (H.P.K.); 2 Chair of Food and Bioprocess Engineering, Technical University of Munich, 85354 Freising, Germany; ulrich.kulozik@tum.de

**Keywords:** β-lactoglobulin, α-lactalbumin, whey proteins, denaturation, reactivity, thermomechanical treatment, protein mixing ratio, phase separation

## Abstract

The influence of thermomechanical treatment (temperature 60 °C–100 °C and shear rate 0.06 s^−1^–50 s^−1^) and mixing ratio of β-lactoglobulin (βLG) and α-lactalbumin (αLA) (5:2 and 1:1) on the denaturation and aggregation of whey protein model systems with a protein concentration of 60% and 70% (*w/w*) was investigated. An aggregation onset temperature was determined at approx. 80 °C for both systems (5:2 and 1:1 mixing ratio) with a protein concentration of 70% at a shear rate of 0.06 s^−1^. Increasing the shear rate up to 50 s^−1^ led to a decrease in the aggregation onset temperature independent of the mixing ratio. By decreasing the protein concentration to 60% in unsheared systems, the aggregation onset temperature decreased compared to that at a protein concentration of 70%. Furthermore, two significantly different onset temperatures were determined when the shear rate was increased to 25 s^−1^ and 50 s^−1^, which might result from a shear-induced phase separation. Application of combined thermal and mechanical treatment resulted in overall higher degrees of denaturation independent of the mixing ratio and protein concentration. At the conditions applied, the aggregation of the βLG and αLA mixtures was mainly due to the formation of non-covalent bonds. Although the proportion of disulfide bond aggregation increased with treatment temperature and shear rate, it was higher at a mixing ratio of 5:2 compared to that at 1:1.

## 1. Introduction

Extrusion processing has been used to produce functional whey protein-based emulsifiers and thickeners [1,2,3,4]. During extrusion processing, highly concentrated whey proteins (concentrations above 30%) are treated simultaneously thermally and mechanically, which can lead to protein denaturation and the formation of structures consisting of aggregated proteins. Depending on the treatment conditions (thermal and mechanical stress profile, and milieu conditions), the globular protein structures unfold, new protein–protein interactions are formed, and aggregation takes place. Since the properties of the resulting aggregates (e.g., form, size, and stabilizing intermolecular interactions) play a crucial role in the functionality of the treated proteins [5], controlling the final product properties requires exact data on the reactions taking place (i.e., denaturation and aggregation).

The main whey protein fractions are β-lactoglobulin (βLG) and α-lactalbumin (αLA). There is plenty of information about the effect of temperature and/or shear rate on the denaturation of whey proteins at concentrations below 10%. βLG is predominantly present as a dimer (D) in solutions at 25 °C and neutral pH [6,7]. Increasing the temperature leads to various conformational changes including a shift in the equilibrium towards the monomeric form [8] and exposure of free thiol groups and hydrophobic patches at around 40 °C [9]. At temperatures above 65 °C, the exposed thiol groups of the βLG monomers react with those accessible through the unfolding disulfide bonds, which results in a thiol–disulfide exchange reaction [10] and eventually in protein aggregation. In contrast to βLG, αLA in solution is very heat-stable with respect to aggregation, due to its four stabilizing disulfide bonds and no free thiol group [11]. Although thermal denaturation of αLA begins at lower temperatures compared to βLG (approx. 35 °C), it is completely reversible up to a high temperature range [12]. Only treatment at temperatures ≥100 °C of αLA is known to result in disulfide bond loss leading to irreversible aggregation via the thiol–disulfide exchange reaction [13,14]. In contrast, in presence of other fractions, αLA participates in the aggregation with βLG monomers via the described thiol–disulfide exchange at lower temperatures (75 °C) as shown in [15].

In our recent study [16], the influence of thermomechanical treatment on the denaturation of whey proteins (αLA, βLG, and whey protein isolate (WPI)) was investigated at a protein concentration of 70% (*w/w*) (unpublished data). The results showed that a combination of thermal and mechanical treatment led to higher degrees of denaturation for all the protein systems investigated. In contrast to many studies, αLA showed higher degrees of denaturation compared to WPI and βLG even without the additional influence of shear stress. Although the denaturation reaction order for αLA and βLG was very similar (2.262 and 2.151, respectively), for WPI systems, a significantly different reaction order of 1.865 was determined. Additionally, the results showed that the shear stress influenced the denaturation reaction of βLG and αLA differently. These results show that the denaturation and aggregation kinetics known from diluted systems cannot be simply applied for highly concentrated systems. Firstly, because depending on protein concentration, the effect of individual process parameters is expected to differ significantly. Moreover, as previously shown in [17,18] the reaction behavior changes with concentration, which might explain the increase in reaction order from 1.5 (concentration <20%) to 1.909–2.262 (concentration >50%). Since the reaction behaviors of βLG and αLA differed from the reaction behavior of mixed systems (WPI), it is expected that changes in the concentration and ratio of βLG and αLA lead to changes in the denaturation and aggregation behavior of complex systems such as WPI. 

Depending on the source of whey (i.e., sweet whey; acid whey; WPI derived from milk protein fractionation by microfiltration without acidification or renneting) and applied extraction/purification method, protein powders with different compositions such as ratio and concentration of βLG and αLA, ionic milieu, and other constituents such as caseinomacropeptide (CMP; in case of sweet/rennet whey) are obtained. Since such protein powders are used to produce functional ingredients via extrusion processing, understanding the influence of the ratio of main protein fractions on the thermomechanical denaturation and aggregation of whey proteins at high concentrations is needed to eventually be able to control the reactions and by this, the properties of the resulting products. Therefore, the objective of this study was to investigate the effect of thermal and mechanical treatment and mixing ratio of βLG and αLA on the denaturation of highly concentrated whey proteins. βLG and αLA mixing ratios of 5:2 and 1:1 were chosen. The mixing ratio of 5:2 was chosen since this is the natural occurrence of each fraction in whey. In order to be able to investigate the effect of a lower relative amount of βLG on the reactions between βLG and αLA, a mixing ratio of 1:1 was applied. In addition to the influence of the mixing ratio, the influence of temperature, shear rate, and protein concentration on the reaction onset temperature of mixed systems was also investigated. Furthermore, the degree of denaturation and aggregation for the chosen whey protein systems was analyzed.

## 2. Materials and Methods

### 2.1. Materials

Pure bovine βLG was produced at the Chair of Food and Bioprocess Engineering of the Technical University Munich at Freising-Weihenstephan, Germany. The βLG powder used in our previous works [17,19,20] was isolated from whey protein solutions as described by [21]. The previously used lot of βLG was produced using water de-hardened by ion exchange, and it therefore had an ionic strength sufficient to stabilize the pH of the solution with distilled water as rehydration medium at pH 6.25. The βLG used in this study was isolated using deionized water as the diafiltration medium, i.e., at very low ionic strength. Therefore, the βLG redissolved in distilled water had a pH of 4.77 due to the missing buffering capacity of the aqueous phase. The method is described in [22]. The βLG was highly pure (<0.05% lactose, minerals <0.13%), the moisture content was <4%, the degree of nativity was >99%, with 96.8% protein on dry matter, the content of βLG in the dry matter was >99%. Alpha-lactalbumin (αLA), BiPro Alpha 9000, was kindly provided by Agropur Inc. (Agropur, Wisconsin, USA). According to the supplier, the αLA used in this study was highly native and pure (<0.2% lactose), the moisture content was <5.6%, with 97.6% protein on dry matter, and the content of αLA in the dry matter was 92.2%.

### 2.2. Sample Preparation

βLG and αLA were mixed with deionized water (Millipore Sigma, Burlington, MA, USA) to produce protein doughs with a protein concentration of 60% and 70% (*w/w*) with a Thermomix (Vorwerk, Wuppertal, Germany) for 3 min. Two different model samples were prepared by mixing βLG and αLA to achieve a mixing ratio of 5:2 and 1:1 (*w/w*). The specific composition of each sample is displayed in Table 1. To ensure a homogenous distribution of water, the samples were stored at 8 °C for at least two days before any experiment.

### 2.3. Thermomechanical Treatment and Inline Rheological Analyses

Thermomechanical treatment and rheological measurements were performed by a closed-cavity rheometer CCR (RPA flex, TA Instruments, New Castle, DE, USA) as previously described in detail in [19]. The test chamber consists of two cones in opposite. Both geometries are grooved to prevent slippage and are thermoregulated by direct heating and forced air-cooling. The test chamber is sealed and pressurized at 0.6 MPa to prevent water evaporation during the experiments. Additionally, high torque levels (up to 25 Nm) can be realized, which are necessary to process the highly viscous protein systems. The mechanical treatment (i.e., oscillatory shear) was applied through the oscillation of the lower cone with a defined frequency and strain. The dynamic shear rates are calculated as shown (Equation (1)) from a combination of angular frequency 2π*f* and absolute strain $\gamma_{abs}$, which is calculated from the angular displacement (strain γ and $\frac{\pi }{180}$) and a strain constant $\frac{1}{2\theta_{cone}}$, with $\theta_{cone}$ as the cone angle (Equation (2)).

(1) $$\dot{\gamma }=2*\pi *f*\gamma_{abs}$$

(2) $$\gamma_{abs}=\gamma *\frac{\pi }{180}*\frac{1}{2*\theta_{cone}}$$

For the determination of the reaction onset temperatures, temperature sweep analyses within a temperature range of 30 °C–180 °C were carried out in triplicate. The measurements were performed at a heating rate of 5 K min^−1^ with a frequency of 1 Hz and strain of 0.07°, which correspond to the linear viscoelastic (LVE) region. Additionally, to investigate the influence of time, temperature, and shear rate on the reactions taking place, isothermal time sweep measurements were conducted. The effect of mechanical treatment at a dynamic shear rate of 0.06 s^−1^ can be neglected, as this shear rate corresponds to the LVE region of the samples. Samples were treated with a treatment temperature of 60, 70, 80, 90, and 100 °C, with shear rates of 0.06, 25, and 50 s^−1^, and treatment times up to 90 s, respectively. It should be noted that due to the small volume of measuring chamber (4.5 cm^3^) and pre-heated lower and upper geometries, the samples achieved the chosen treatment temperatures almost instantaneously. Even though during the thermomechanical treatments, the temperature of the chamber geometry is controlled by electric heating and forced air-cooling, at very high protein concentrations and shear rates, viscous dissipation energy led to slightly higher chamber temperatures than the set temperatures. Therefore, the temperatures in the results section are given as a mean value with standard deviation to include this effect. After thermal and mechanical treatment, the samples were dried in a vacuum dryer (Heraeus, Hanau, Germany) at 25 °C and 10 mbar and milled with a coffee mini grinder (KYG Group, Dongguan, China) to a particle size < 500 µm. For further offline analyses, only the dried protein powders were used. All measurements were conducted at least in duplicate.

### 2.4. Degree of Denaturation

The amount of native protein remaining in solution after the treatment was measured using UV–Vis spectroscopy using an Evolution 201 spectrophotometer (Thermo Fischer Scientific Inc., Waltham, MA, USA) to determine the degree of denaturation. To induce precipitation of denatured proteins, the CCR-treated and milled samples were dissolved in acetate buffer (0.5 M, pH 4.6) at a concentration of 1 mg mL^−1^. After an extraction time of one hour, the denatured proteins aggregated during the foregoing treatment in the closed-cavity rheometer were removed by centrifugation at 4301× *g* for 60 min; ensuring that only soluble, and by this, native fractions remained in the supernatant. Absorption of the samples was measured at a wavelength of 280 nm. Untreated samples were used as reference, and every extraction was performed in duplicate for each sample. Using a calibration curve, the concentration in the supernatant was calculated from the measured absorption. The degree of denaturation (*D_D_*) (Equation (3)) was calculated as the protein concentration ratio after thermomechanical treatment (*C_T_*) and that before treatment (*C*_0_).

(3) $$D_{D}=\left(\left(\frac{C_{0}-C_{T}}{C_{0}}\right)*100\%\right)$$

### 2.5. Analysis of Protein–Protein Interactions Leading to Aggregation

To investigate which protein–protein interactions between βLG and αLA molecules are responsible for the aggregation of the treated samples, the solubility of the samples in various buffer solutions cleaving specific bonds was analyzed as previously described in detail in [17].

For the analysis of the participation of non-covalent (i.e., electrostatic interactions, hydrophobic, and hydrogen bonds) and covalent bonds (i.e., disulfide bonds and isopeptide bonds) in the aggregation of model systems after thermal and mechanical treatment, treated and untreated samples were solved in three 0.02 M phosphate buffers at pH 7.0. The phosphate buffer (Buffer 1) contained only phosphate salts and was used to set a defined and constant pH of 7.0 for all solutions. The phosphate buffer containing 0.05 M NaCl, 0.03 M SDS, and 8 M urea, was used to investigate the participation of covalent bonds, as the chemicals used are able to cleave or prevent the formation of new electrostatic interactions, hydrogen, and hydrophobic bonds. Adding 0.03 M DTT to this buffer, results in the cleavage of disulfide bonds. By this, information on the participation of other covalent bonds beside disulfide bonds is gained. These extraction conditions are called non-reducing and reducing conditions, respectively. For the analysis, samples at a concentration of 1 mg mL^−1^ were prepared with the three buffers. After 24 h of extraction on a rotary shaker at 200 rpm, insoluble proteins were removed by centrifugation at 4301× *g* for 60 min; ensuring only soluble proteins remained in the supernatant. Formerly aggregated proteins (insoluble proteins), formed through either non-covalent (e.g., hydrophobic interactions), covalent (e.g., disulfide and isopeptide bonds), or only disulfide bonds in the thermomechanical treatment step in the CCR device were de-aggregated and thus made soluble through cleaving the participating bonds and interactions. The supernatant was then analyzed for protein solubility using UV–Vis spectroscopy at a 280 nm detection wavelength. Untreated samples were also measured as a reference. The measurements were performed in duplicate for each sample. For each mixing ratio (5:2 and 1:1), a calibration curve was plotted to be able to determine the concentration in the supernatant calculated from the measured absorption. The degree of non-covalent aggregation (*DA_nC-B_*) (Equation (4)) was calculated from the difference of the initial concentration of untreated sample (*C*_0_) with the residual protein remaining in the supernatant (*C_T_*) after the extraction using Buffer 1.

(4) $$DA_{nC-B}=\left(C_{0}-C_{t}\right)|Buffer1$$

(5) $$DA_{C-B}=\left(C_{0}-C_{t}\right)|non-reducing$$

(6) $$DA_{nS-B}=\left(C_{0}-C_{t}\right)|reducing$$

(7) $$DA_{S-B}=A_{C-B}-A_{nS-B}$$

The degrees of covalent bond aggregation (*DA_C−B_*) (Equation (5)) and non-disulfide covalent bond aggregation (*DA_nS−B_*) (Equation (6)) were calculated similarly from the difference of the initial concentration of untreated sample with the residual protein remaining in the supernatant after the extraction under non-reducing conditions and reducing conditions, respectively. The degree of disulfide bond aggregation (*DA_S−B_*) (Equation (7)) was calculated from the difference between Equations (5) and (6).

### 2.6. Statistical Analysis

All experiments were performed at least in duplicate. Mean values and standard deviations are reported.

## 3. Results and Discussion

### 3.1. Influence of Thermomechanical Treatment on the Reaction Behavior and Onset Temperatures of Model Samples

**Figure 1:**
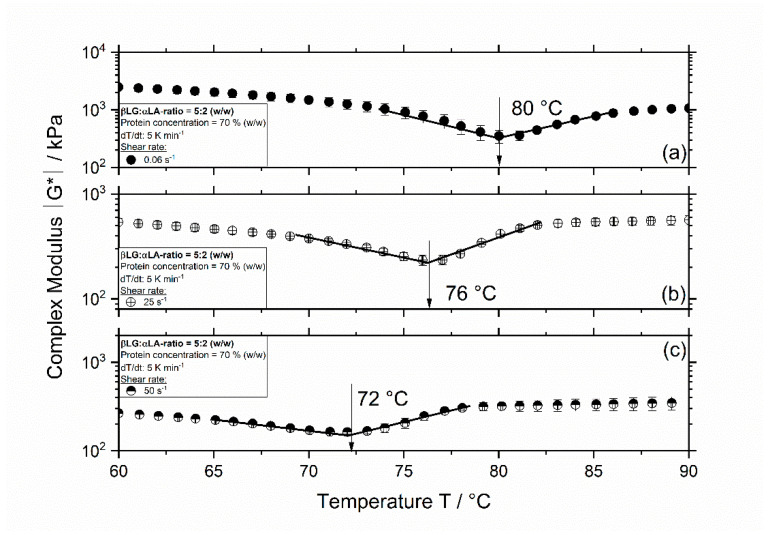
Complex modulus $\left|G^{*}\right|$ as a function of temperature for 5:2 systems with a concentration of 70% (*w/w*) and a shear rate of 0.06 s^−1^ (**a**), 25 s^−1^ (**b**), and 50 s^−1^ (**c**). Measurements were performed at a constant heating rate of 5 K min^−1^, bars represent standard deviation (SD).

The complex modulus $\left|G^{*}\right|$ is depicted in Figure 1 as a function of temperature and shear rate for the system containing a mixing ratio of 5:2 (βLG:αLA) and a protein concentration of 70% (*w/w*).

The complex modulus in Figure 1a shows an initial decrease (Region I) followed by an increase (Region II). The initial decrease in the complex modulus was expected, as by increasing the temperature the molecular mobility also increases leading to decreased values of viscosity, and by this of complex modulus. Thereafter, a distinct change in the slope of the curve is observed at approx. 80 °C (transition from Region I and II), which is defined as the aggregation onset temperature [19,23]. Although the aggregation might have started at lower temperatures, this is the temperature where the aggregation outweighs the effect seen in Region I. Even though the curve describing the course of the complex modulus remained unchanged when the shear rate is increased from 0.06 to 25 and 50 s^−1^, the aggregation onset temperature decreased. At a shear of 25 s^−1^ (Figure 1b) and 50 s^−1^ (Figure 1c), the aggregation onset temperature was approx. 76 and 72 °C, respectively.

**Figure 2:**
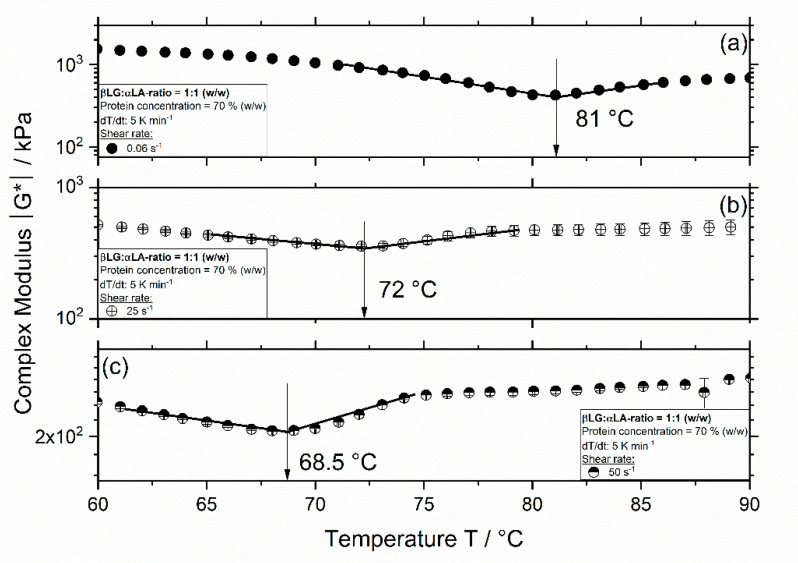
Complex modulus $\left|G^{*}\right|$ as a function of temperature for 1:1 systems with a concentration of 70% (*w/w*) and a shear rate of 0.06 s^−1^ (**a**), 25 s^−1^ (**b**), and 50 s^−1^ (**c**). Measurements were performed at a constant heating rate of 5 K min^−1^, bars represent standard deviation (SD).

In Figure 2, the course of the complex modulus for the sample containing same amounts of βLG and αLA (mixing ratio 1:1) is depicted as a function of temperature and shear rate. Similar to the results in Figure 1, the complex modulus curve shows two distinct regions, and an aggregation onset temperature is observed at approx. 81, 72, and 68.5 °C for a shear rate of 0.06, 25, and 50 s^−1^, respectively. For both systems, independently of the mixing ratio of βLG and αLA, an increase in shear rate leads to a decrease in the aggregation onset temperature. Furthermore, the determined onset temperatures are lower for systems containing a mixing ratio of 1:1 compared to those for the 5:2 systems.

The decrease in the aggregation onset temperature with increase shear rate arises probably due to the increased molecular mobility at higher shear rates resulting in a lower energies needed for reactions to take place (i.e., activation energy decreases) leading to lower onset temperatures. For protein aggregation to occur, it is known that the bonds stabilizing the native structure break up (unfolding), and then new bonds are formed leading to aggregation. In this case, the shear rate might induce conformational changes, i.e., partial unfolding, which has a higher energy level in the reaction coordinate. Therefore, it is then expected that the energy barrier (unfolding + formation new bonds) is lower, and the activation energy is also lower. Accordingly, the decrease in reaction onset temperature with shear rate has been already observed in our previous study for βLG and αLA (i.e., single fractions) and whey protein isolate [16]. The decrease in the aggregation onset temperature for 1:1 systems was expected since the aggregation of βLG is enhanced when αLA is present. This has already been observed for more dilute systems [15,24] and at higher protein concentrations [16]. αLA denatures at lower temperatures compared to βLG, but since in its native form no free thiol group is available, therefore, most of the aggregation takes place by the formation of non-covalent bonds, resulting in weaker aggregates. In combined systems, βLG initiates the aggregation and contributes with a highly reactive free thiol group, which could result in the aggregation of βLG and αLA via disulfide bonds due to a thiol–disulfide exchange reaction.

Previous results have shown that shearing of systems containing multiple protein fractions can lead to phase separation [16,25]. Although [25] observed no phase separation for whey protein concentrate (WPC) gels with a protein concentration of 7% [25], phase separation is indeed expected at higher concentrations due to the thermodynamic incompatibility resulting from the limited solubility of the biopolymers involved [26]. Phase separation of mixed biopolymer solutions involves either segregation of the each biopolymer into individual phases or the separation of some coacervated concentrated phase involving both biopolymers. From a thermodynamic point of view, mixing different (bio-)polymers results in a limited entropy gain, therefore, enthalpic effects tend to promote demixing and, consequently, the separation of each biopolymer in solution [27]. In [16], phase separation induced by shearing was hypothesized since during temperature sweep analysis without shear, a single aggregation temperature was determined for whey protein isolate (WPI) at a concentration of 70%. In contrast, during combined shearing and temperature sweep analysis, these tests led to two onset temperatures, corresponding to the main protein fractions present (βLG and αLA, respectively) [16].

**Figure 3:**
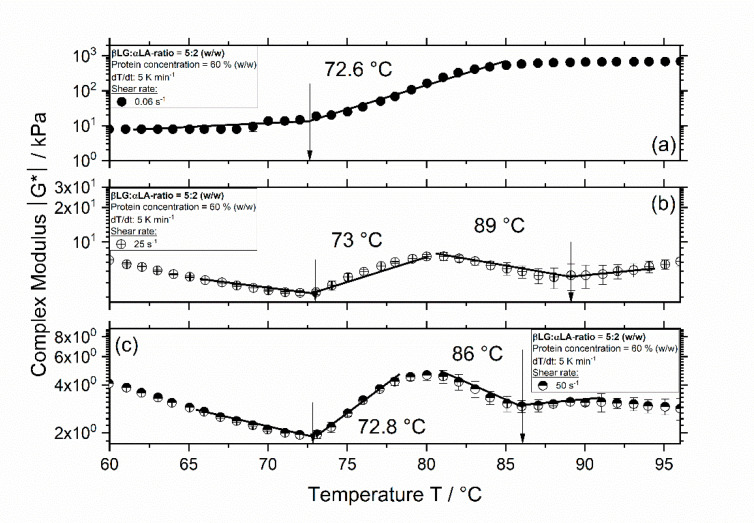
Complex modulus $\left|G^{*}\right|$ as a function of temperature for 5:2 systems with a concentration of 60% (*w/w*) and a shear rate of 0.06 s^−1^ (**a**), 25 s^−1^ (**b**), and 50 s^−1^ (**c**). Measurements were performed at a constant heating rate of 5 K min^−1^, bars represent standard deviation (SD).

In contrast to the results of WPI at a concentration of 70%, phase separation occurs in 5:2 systems at a lower protein concentration of 60% (Figure 3). In Figure 3a, the system with concentration of 60% (*w/w*) without the influence of shear rate, shows only one aggregation onset temperature. Compared to the same system at higher protein concentration (Figure 1a), the aggregation onset temperature is lower since it decreases from approx. 80 to 73 °C. At a shear rate of 25 s^−1^, two different onset temperatures are observed at approx. 73 and 89 °C. Increasing the shear rate to 50 s^−1^, leads to a slight decrease in the aggregation onset temperatures to 72.8 and 86 °C. Similarly, for 1:1 systems, two onset temperatures were determined when the systems were sheared, and a decrease in protein concentration from 70% and 60% resulted in a decrease in aggregation onset temperatures (data not shown). Since these systems showed a similar behavior, only the results from 5:2 systems are shown.

**Figure 4:**
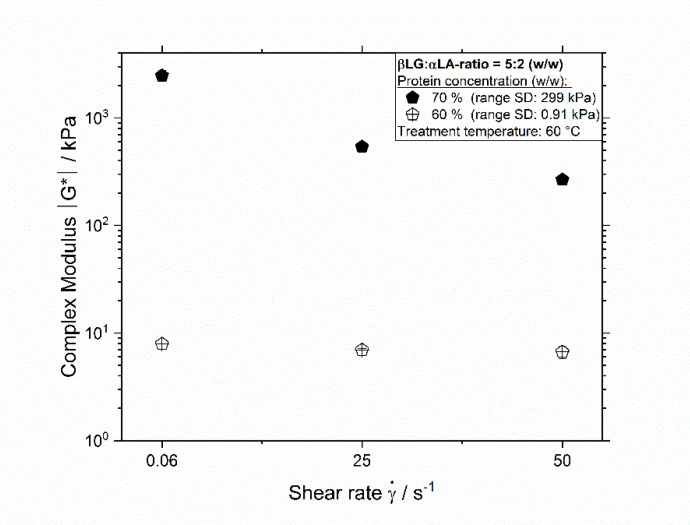
Complex modulus $\left|G^{*}\right|$ of 5:2 systems in dependence of the shear rate for samples treated at 60 °C with a protein concentration of 60% and 70% (*w/w*), bars represent standard deviation (SD).

The decreasing onset temperature of highly concentrated systems with increasing shear rate is expected to arise from an increase in molecular mobility with increasing the shear rate, which is characterized by a decrease in viscosity, and by this, in the complex modulus. As shown by the results in Figure 4, the values of the complex modulus for a protein concentration of 60% are not significantly different when the shear rate increases from 0.06 to 50 s^−1^ at 60 °C. Therefore, for this system, the maximum enhancing effect on the reactions is expected in this shear rate range, which results in very similar onset temperatures even when the shear rate increases from 25 to 50 s^−1^. In contrast, at 70%, the values of the complex modulus at different shear rates are significantly different, which indicates that the shear stress influences the molecular motion and, thus, the reaction rate significantly. It has been shown for whey proteins that an increase in protein concentration leads to a decrease in molecular mobility and, consequently, in higher values of activation energy, which result in higher onset temperatures [18,19]. By increasing the shear rate, the molecular motion increases, and the reactions activation energy decreases, which leads to lower onset temperatures [16,17]. Comparing the values of the complex modulus in Figure 4, it can be seen that the values of the complex moduli are almost two orders of magnitude higher when the concentration increases from 60% to 70% (*w/w*), implying a decrease in the reaction rate. Although, for systems with a protein concentration of 70%, the shear rate significantly influences the complex modulus and by this the molecular mobility, which should lead to higher reaction rates, it seems that the increased molecular interaction between molecules resulting from oscillatory shear flow is not enough to induce phase separation. Since phase separation increases with increasing protein denaturation, it seems plausible that at the higher protein concentration of 70%, where the molecular mobility is low and, therefore, both of the main fractions have a similar high onset temperature, no phase separation is detected. At higher shear rates, the negative effect of the protein concentration on the reactions decreases, making molecules to denature at lower temperatures and aggregate at faster rates, which consequently results in phase separation.

### 3.2. Influence of Thermomechanical Treatment on the Degree of Denaturation

**Figure 5:**
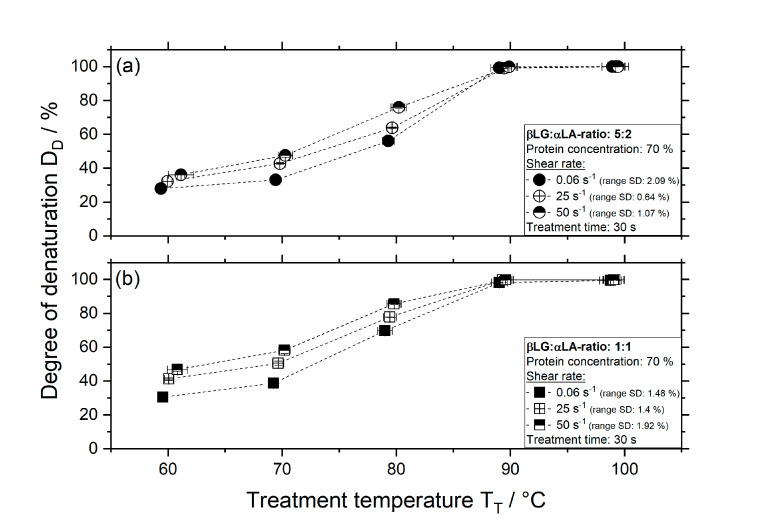
Degree of denaturation as a function of temperature and shear rate for 5:2 systems (**a**) and 1:1 systems (**b**) with a protein concentration of 70% (*w/w*) and treatment time of 30 s, bars represent standard deviation (SD). The range of SD for the treatment temperature was for all measurements ≤ 1.1%.

The influence of treatment temperature and shear rate on the *D_D_* for samples containing a mixing ratio of 5:2 and 1:1 βLG and αLA at a concentration of 70% (*w/w*) treated for 30 s is depicted in Figure 5.

As shown in Figure 5a, thermal treatment of the 5:2 system results in a degree of denaturation of 28% and 33% for 60 and 70 °C, respectively. The degree of denaturation for samples treated thermally at 80 °C is approx. 56%.

At higher temperatures, the degree of denaturation is already above 90%, even at the lower shear rate of 0.06 s^−1^. Increasing the shear rate to 50 s^−1^, leads to an increase in denaturation for all temperatures investigated. Thermomechanical treatment of 5:2 samples at a shear rate of 50 s^−1^ and temperatures of 60, 70, and 80 °C results in a degree of denaturation of approx. 36%, 48%, and 76%, respectively. At temperatures above 90 °C, the effect of the shear rate on the denaturation is no longer visible as at these conditions the denaturation reaction is already high.

Thermal treatment of 1:1 samples at a concentration of 70% (Figure 5b) results in slightly higher degrees of denaturation compared to the 5:2 systems. Treatment at 60, 70, and 80 °C results in a degree of denaturation of approx. 30%, 39%, and 70%, respectively. Thermomechanical treatment at a shear rate of 50 s^−1^ and temperatures of 60, 70, and 80 °C results in a degree of denaturation of approx. 47%, 58%, and 86%, respectively.

From the results shown in Figure 1 and Figure 2, it was indeed expected that the 1:1 system shows a higher degree of denaturation since the onset temperature was lower for this system compared to that of the 5:2 system. In contrast, the high degree of denaturation (above 20%) at 60 °C independent of the mixing ratio and shear rate was not expected. Although in our previous study [16], it was shown that the denaturation of 70% αLA was higher compared to that in the studies at lower concentrations (10%), thermal treatment at 60 °C resulted in approx. 7% denaturation. The increase in denaturation to approx. 30% for 5:2 and 1:1 systems could be explained by the presence of highly pure and native βLG in the mixed systems. Although a mixing ratio of 5:2 is similar to the protein ratio of βLG and αLA in WPI, previous results reported on Quevedo et al. (2021b) showed that the degree of denaturation of 70% WPI was approx. 2% at a treatment temperature of 60 °C and 0.06 s^−1^ [16]. Therefore, the high degree of denaturation depicted in Figure 5 could also result from the absence of lactose in the mixed systems, which is known to have a hindering effect on the denaturation of whey proteins [28,29].

### 3.3. Influence of Thermomechanical Treatment on the Protein–Protein Interactions Leading to Aggregation

**Figure 6:**
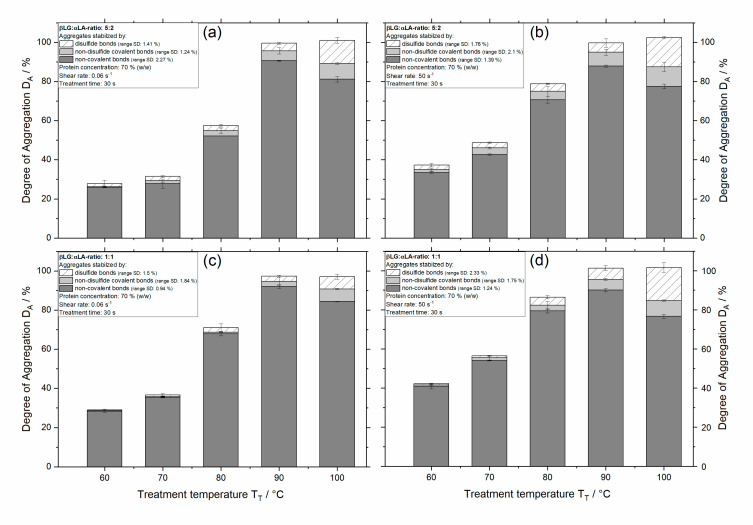
Degree of aggregation as a function of temperature for 5:2 systems at a shear rate of 0.06 s^−1^ (**a**), 50 s^−1^ (**b**), and 1:1 systems at a shear rate of 0.06 s^−1^ (**c**) and 50 s^−1^ (**d**) with a protein concentration of 70% (*w/w*) and a treatment time of 30 s. The solid dark grey portion of the bar gives the proportion of stabilization by non-covalent bonds, that by non-disulfide covalent bonds is given by the solid light grey portion of the bar, and that by disulfide bonds is given by the hatched portion of the bar, bars represent standard deviation (SD).

The degree of aggregation caused by non-covalent and covalent bonds (i.e., disulfide bonds and non-disulfide covalent bonds such as isopeptide bonds or lysinoalanine or lanthionine) as a function of treatment temperature and shear rate for 5:2 and 1:1 systems at a concentration of 70% (*w/w*) and 30 s is depicted in Figure 6. Similar to the results of the degree of denaturation, the aggregation also increases with increasing treatment temperature and shear rate. Independently of the mixing ratio or shear rate applied, treatment at temperatures of 90 °C and above results in 100% aggregation. Additionally, the 1:1 system shows slightly higher degrees of aggregation.

Regarding the formation of new protein–protein interactions, it is shown that for both systems the proportions of newly formed non-covalent bonds outweighs that of the covalent bonds. Furthermore, as shown in Figure 6a,b more aggregation is taking place via disulfide bonds for the 5:2 systems, compared to that for the 1:1 system (Figure 6c,d). In contrast, the 1:1 system seems to aggregate mainly via non-covalent bonds, which appears to be meaningful, since the lower relative amount of βLG provides fewer available thiol groups.

Increasing the treatment temperature leads to more aggregation, firstly non-covalent bonds are formed, then when the temperature is high enough (100 °C), there is a shift in the formation of interactions, and aggregation takes place via disulfide bonds. Comparing the samples treated at 90 and 100 °C, the degree of non-covalent bond aggregation decreases, and the disulfide bond aggregation increases further. Although the formation of non-covalent bonds was expected, since it is known that treatment below 80 °C of αLA and βLG in milk samples results mostly in non-covalent bond aggregation [30], it was not expected that it outweighed the formation of disulfide bonds at the treatment temperatures investigated. In [20], the aggregation behavior of βLG at concentrations above 50% was investigated, and it was shown that the aggregation between βLG molecules was mainly via disulfide bonds and non-disulfide covalent bonds. Additionally, thermal treatment of 70% αLA (*w/w*) at 80 and 100 °C resulted in a degree of disulfide bond aggregation of 30% and 85%, respectively (data not shown). Since covalent bond aggregation was at least 30% for the single protein fractions, a similar behavior was expected for combined systems.

In contrast to the βLG used in previous studies [17,19,20], as already mentioned in the materials section, in this study a different lot of βLG powder was used, which was probably the cause of the observed aggregation behavior in these mixed systems. Due to the low ionic strength and, consequently, low buffering capacity of the solvent used during the purification step, a βLG lot with rather low pH (~4.7) was produced. Since the reactivity of the thiol groups decreases with decreasing pH, the high degree of non-covalent bond aggregation probably may arise from this occurrence. Accordingly, previous results showed that the variation of pH had a considerable effect on the denaturation reactions [18]. Regarding the aggregation behavior and the influence of pH on the formation of protein–protein interactions, it is known that for whey protein systems at concentrations below 10%, at pH <7 the thiol groups become less reactive and that non-covalent bonds are increasingly being formed leading to aggregates stabilized via non-covalent and disulfide bonds [31]. Due to the pH-induced increase in positive charge of the proteins’ surface occurring at low pH values [32], strong repulsive forces may arise, which then prevent the formation of disulfide bonds. Since the βLG used for the mixed systems investigated in this study had a very low ionic strength, it is possible that even small differences in charge lead to a longer range of the electrical double layer, which could have a similar effect to very low pH values resulting in less disulfide bonds being formed.

## 4. Conclusions

The influence of thermomechanical treatment and mixing ratio of βLG and αLA on the denaturation and aggregation of whey proteins was investigated at protein concentrations of 60% and 70% (*w/w*). Results of temperature sweep analysis showed that the aggregation onset temperatures were lower for systems containing a mixing ratio of βLG and αLA of 1:1 compared to those of 5:2 systems. Furthermore, independently of the mixing ratio of βLG and αLA, increasing the shear rate led to a decrease in the aggregation onset temperature. Similarly, and also independently of the mixing ratio, decreasing the protein concentration from 70% to 60% resulted in a decrease of the onset temperatures. At a protein concentration of 60%, shearing of the systems induced phase separation since two onset temperatures were determined for a shear rate of 25 and 50 s^−1^. Although phase separation is also expected at higher concentrations, with the applied measuring method, no phase separation was observed at a concentration of 70% even at 50 s^−1^. Since phase separation is expected to increase with denaturation, at high concentrations, it will occur at higher shear rates, when molecular mobility increases and the activation energy is reduced, compared to lower concentrations. A combination of thermal and mechanical treatment led to higher degrees of denaturation and aggregation for both model systems investigated, whereas the 1:1 systems showed overall slightly higher values compared to those of the 5:2 systems. Furthermore, the aggregation behavior of mixed model systems was mainly due to the formation of non-covalent bonds. Although the proportion of the disulfide bond aggregation increased with treatment temperature and shear rate, the degree of aggregation via disulfide bonds was higher for the 5:2 systems compared to that of the 1:1 systems. Therefore, depending on the pH/ionic strength and mixing ratio of the proteins present in the protein system, the aggregation pathway of whey proteins can be changed. At the investigated conditions, aggregation took place mainly via non-covalent bonds, which could lead to improved functionalities. Since aggregates stabilized via strong covalent bonds lose their interfacial functionality due to the lacking ability of unfolding at the interfaces (oil/water or air/water), aggregates stabilized via weaker non-covalent bonds might still be able stabilize emulsions or foams. This information forms the basis for a better understanding of the denaturation and aggregation reaction behavior of highly concentrated whey proteins at extrusion-like conditions and represents the first step towards being able to control a complex process such as extrusion processing.

## Figures and Tables

**Table 1:** Concentration of main whey protein fraction in model samples with a total protein concentration of 60% and 70% (*w/w*).

βLG:αLA Mixing Ratio	Protein Concentration/%	Total Mass Sample/g	βLG/g	αLA/g	Deionized Water/g
1:1	70	100	35.02	35.02	29.96
5:2			50.08	19.96	29.96
1:1	60	100	30.02	30.02	39.96
5:2			42.93	17.11	39.96

βLG—beta-lactoglobulin; αLA—alpha-lactalbumin.

## References

[1] Koch L., Hummel L., Schuchmann H.P., Emin M.A. (2018). Improving the emulsifying properties of whey protein isolate-citrus pectin blends by a novel reactive extrusion approach. J. Food Eng..

[2] Manoi K., Rizvi S.S.H. (2008). Rheological characterizations of texturized whey protein concentrate-based powders produced by reactive supercritical fluid extrusion. Food Res. Int..

[3] Queguiner C., Dumay E., Salou-Cavalier C., Cheftel J.C. (1992). Microcoagulation of a whey protein isolate by extrusion cooking at acid pH. J. Food Sci..

[4] Wolz M., Kastenhuber S., Kulozik U. (2016). High moisture extrusion for microparticulation of whey proteins—Influence of process parameters. J. Food Eng..

[5] Dissanayake M., Liyanaarachchi S., Vasiljevic T. (2012). Functional properties of whey proteins microparticulated at low pH. J. Dairy Sci..

[6] McKenzie H.A., Sawyer W.H. (1967). Effect of pH on beta-lactoglobulins. Nature.

[7] Verheul M., Roefs S.P.F.M., Kruif K.G.d. (1998). Kinetics of heat-induced aggregation of β-lactoglobulin. J. Agric. Food Chem..

[8] Georges C., Guinand S., Tonnelat J. (1962). Etude thermodynamique de la dissociation réversible de la β-lactoglobuline B pour des pH supérieurs à 5,5. Biochim. Et Biophys. Acta.

[9] Loveday S.M. (2016). β-Lactoglobulin heat denaturation: A critical assessment of kinetic modelling. Int. Dairy J..

[10] Hoffmann M.A.M., Mil P.J.J.M.V. (1997). Heat-induced aggregation of β-lactoglobulin: Role of the free thiol group and disulfide bonds. J. Agric. Food Chem..

[11] Sienkiewicz T. (1981). Nomenklatur und einige eigenschaften der molkenproteine. 2. Mitt. α-Lactalbumin, immunoglobuline, proteose-peptone, minorproteine und enzyme. Nahrung.

[12] Apenten R.K.O. (1995). Thermodynamic parameters for 3-state thermal denaturation of human and bovine α-lactalbumin. Thermochim. Acta.

[13] Chaplin L.C., Lyster R.L.J. (1986). Irreversible heat denaturation of bovine α-lactalbumin. J. Dairy Res..

[14] Schnack U., Klostermeyer H. (1980). Thermal decomposition of alpha-lactalbumin: I. Destruction of cystine residues. Milchwissenschaft.

[15] Schokker E.P., Singh H., Creamer L.K. (2000). Heat-induced aggregation of β-lactoglobulin A and B with α-lactalbumin. Int. Dairy J..

[16] Quevedo M., Karbstein H.P., Emin M.A. (2020). Denaturation behavior and kinetics of single- and multi-component protein systems at extrusion-like conditions. Foods.

[17] Quevedo M., Kulozik U., Karbstein H.P., Emin M.A. (2020). Kinetics of denaturation and aggregation of highly concentrated β-Lactoglobulin under defined thermomechanical treatment. J. Food Eng..

[18] Quevedo M., Karbstein H.P., Emin M.A. (2020). Influence of thermomechanical treatment and pH on the denaturation kinetics of highly concentrated whey protein isolate. J. Food Eng..

[19] Quevedo M., Jandt U., Kulozik U., Karbstein H.P., Emin M.A. (2019). Investigation on the influence of high protein concentrations on the thermal reaction behaviour of β-lactoglobulin by experimental and numerical analyses. Int. Dairy J..

[20] Quevedo M., Kulozik U., Karbstein H.P., Emin M.A. (2020). Effect of thermomechanical treatment on the aggregation behaviour and colloidal functionality of β-Lactoglobulin at high concentrations. Int. Dairy J..

[21] Toro-Sierra J., Tolkach A., Kulozik U. (2013). Fractionation of α-Lactalbumin and β-Lactoglobulin from whey protein isolate using selective thermal aggregation, an optimized membrane separation procedure and resolubilization techniques at pilot plant scale. Food Bioprocess Technol..

[22] Haller N., Kulozik U. (2019). Separation of whey protein aggregates by means of continuous centrifugation. Food Bioprocess Technol..

[23] Koch L., Emin M.A., Schuchmann H.P. (2017). Reaction behaviour of highly concentrated whey protein isolate under defined heat treatments. Int. Dairy J..

[24] Calvo M.M., Leaver J., Banks J.M. (1993). Influence of other whey proteins on the heat-induced aggregation of α-lactalbumin. Int. Dairy J..

[25] Walkenström P., Hermansson A.-M. (1998). Effects of shear on pure and mixed gels of gelatin and particulate whey protein. Food Hydrocoll..

[26] Tolstoguzov V.B. (1993). Thermoplastic extrusion-the mechanism of the formation of extrudate structure and properties. J. Am. Oil Chem. Soc..

[27] Clark A.H. (1996). Biopolymer gels. Curr. Opin. Colloid Interface Sci..

[28] Tolkach A., Kulozik U. (2005). Effect of pH and T on the reaction kinetic parameters of the thermal denaturation of β-lg. Milchwissenschaft.

[29] Spiegel T., Huss M. (2002). Whey protein aggregation under shear conditions - effects of pH-value and removal of calcium. Int. J. Food Sci. Technol..

[30] Oldfield D.J., Singh H., Taylor M.W., Pearce K.N. (1998). Kinetics of Denaturation and aggregation of whey proteins in skim milk heated in an Ultra-High Temperature (UHT) pilot plant. Int. Dairy J..

[31] Zúñiga R.N., Tolkach A., Kulozik U., Aguilera J.M. (2010). Kinetics of formation and physicochemical characterization of thermally-induced beta-lactoglobulin aggregates. J. Food Sci..

[32] Uhrínová S., Smith M.H., Jameson G.B., Uhrín D., Sawyer L., Barlow P.N. (2000). Structural changes accompanying pH-induced dissociation of the beta-lactoglobulin dimer. Biochemistry.

